# Nucleotide sequence variations, gene expression profiles, and host biomarkers associated with ovine mange caused by *Sarcoptes* and *Psoroptes* mites: implications for diagnosis and disease control

**DOI:** 10.3389/fvets.2026.1902195

**Published:** 2026-09-01

**Authors:** Manal A. Babaker, Mohamed Marzok, Ahmed El Sayed, Tahani M. I. Al-Hazani, Ahmed Ateya, Fatimah S. Alharbi, Elham Mohammed Alzahrani, Rowa K. Zarah, Adel I. Almubarak, Hussein Babiker, Turke Shawaf, Mohammed Al-Rasheed, Rasha Yassin Elkhidr, Zakriya Al Mohamad, Omar A. Al-Jabr, Bassem Elmishmishy

**Affiliations:** 1 Department of Chemistry, Faculty of Science, Majmaah University, Al Majmaah, Saudi Arabia; 2 Department of Clinical Sciences, College of Veterinary Medicine, King Faisal University, Al-Ahsa, Saudi Arabia; 3 Department of Animal Health and Poultry, Animal and Poultry Production Division, Desert Research Center (DRC), Mataryia, Cairo, Egypt; 4 Biology Department, College of Science and Humanities, Prince Sattam bin Abdulaziz University, Al-Kharj, Saudi Arabia; 5 Department of Development of Animal Wealth, Faculty of Veterinary Medicine, Mansoura University, Mansoura, Egypt; 6 Department of Biology, College of Sciences, Taif University, Taif, Saudi Arabia; 7 Department of Biology, College of Science, Princess Nourah bint Abdulrahman University, Riyadh, Saudi Arabia; 8 Department of Microbiology, College of Veterinary Medicine, King Faisal University, Al-Asha, Saudi Arabia; 9 Parasitological Department, Faculty of Veterinary Medicine, Mansoura University, Mansoura, Egypt

**Keywords:** antioxidant, APPs, immunity, molecular diagnostics, *Psoroptes* spp., *Sarcoptes* spp., sheep

## Abstract

**Introduction:**

Ovine mange is a highly contagious skin disease that causes substantial economic losses. This study assessed the prevalence, SNPs, gene expression, serum APPs, and immune and antioxidant markers to elucidate the immunological and oxidative mechanisms of mange in Barki sheep.

**Methods:**

A total of 320 adult Barki ewes were enrolled in the study, comprising 80 clinically affected animals and 240 apparently healthy controls. Blood samples were collected from all animals for the determination of acute-phase proteins, immunological and antioxidant biomarkers, and gene expression analyses of CARD9, CLEC7A, TNFAIP3, IL33, SESN2, ATF4, HSPA1A, and SLC7A11. Only clinically affected sheep underwent parasitological confirmation by microscopic examination of deep skin scrapings collected from active skin lesions to identify mange mites.

**Results:**

Microscopic examination confirmed mange in 25% (80/320) of the ewes, with *Sarcoptes* spp. accounting for 75% and *Psoroptes* spp. for 25% of the cases. Infected sheep showed upregulation of CARD9, CLEC7A, IL33, SESN2, ATF4, and HSPA1A, and downregulation of TNFAIP3 and SLC7A11 (*p* < 0.05), with 14 SNPs differing significantly between the healthy and infected groups. Infected animals showed elevated APPs, pro-inflammatory cytokines, and oxidative stress, with reduced IL-10 levels.

**Conclusion:**

Ovine mange is associated with distinct molecular, immunological, and oxidative stress responses that reflect the host’s reaction to mite infestation. To our knowledge, this is the first study to integrate transcriptional profiling with SNP characterization of *CARD9, CLEC7A, TNFAIP3, IL33, SESN2, ATF4, HSPA1A*, and *SLC7A11* in naturally occurring ovine mange. The identified sequence variants, together with gene expression signatures, provide novel candidate molecular biomarkers for disease susceptibility and may support future genetic selection and precision disease management strategies. Additional studies involving larger and independent sheep populations are needed to validate these findings and determine their potential value in disease susceptibility assessment and genetic improvement programs.

## 1 Introduction

Native to Egypt's northwest coastal region, the Barki sheep breed, named after the Libyan province of Barka, is vital to the way of life of the people who live there (1). With 470,000 sheep, they account for 11% of all sheep in Egypt. This breed is widespread from eastern Libya to the western portion of Alexandria, Egypt. Barki sheep are important in this area because of their exceptional resistance to high temperatures, scarce feed availability, and heat stress (2). Basic information is available regarding Barki ewe productivity and body conformation (3).

Mange is a highly contagious skin disease that affects all farm animals worldwide. Mites, which are ectoparasites, are the cause of this disease. Sheep typically experience two types of mange: *sarcoptic* mange (*Sarcoptes scabiei* var. *ovis* mite infestation) and *psoroptic* mange (*Psoroptes ovis* mite infestation) (4). Intense itching throughout the face, ear pinnae, neck, limbs, or entire body, as well as papules, crusts, excoriations, alopecia, lichenification, thickening, and darkening of the skin, are clinical signs of mite infestation. Anorexia, emaciation, and lethargy cause death in the final stages of the disease (5). Weight loss, skin and wool damage, reduced milk supply, poorer pregnancy rates, weak lambs, higher susceptibility to other infectious diseases, additional food and acaricide costs, labor costs, and death are some of the major economic losses caused by the disease. Moreover, effective ectoparasite control is increasingly challenged by the development of detoxification mechanisms that reduce susceptibility to insecticides and acaricides, emphasizing the need for integrated management strategies and an improved understanding of host–parasite interactions (6). According to Mohamed et al. (4), breeders are also at risk of developing health problems. According to Moroni et al. (7), the disease can spread from sheep to humans via milking or sheep husbandry, making it a significant zoonotic concern. In Egypt, mange infestation has been found in over 20% of agricultural animals throughout several governorates (4, 8–10).

Parasitic infestations exacerbate oxidative stress by stimulating the excessive production of reactive oxygen species (ROS) in host tissues, facilitating parasite survival and contributing to tissue injury and disease progression (11). Consequently, oxidative status serves as a critical indicator of host–pathogen interactions and infection-related health impairment (12). Normal physiological function depends on a tightly regulated redox balance, in which antioxidants protect the cells by neutralizing free radicals (13). Disruption of this balance leads to oxidative stress, resulting in cellular and molecular damage associated with various pathological conditions (14). Animals affected by ectoparasites commonly develop inflammatory skin lesions and hypersensitivity reactions due to excessive generation of free radicals. Altered oxidative stress biomarkers have been documented in mite-infested sheep, goats, camels, buffaloes, and pigs (10, 15–18), respectively. Overall, oxidative stress–mediated tissue damage plays a central role in the pathophysiology of ectoparasitic and dermatological diseases (19).

Cytokines are key regulators of immune and inflammatory responses, functioning as mediators with both pro- and anti-inflammatory effects. They govern immune cell proliferation, differentiation, activation, and maturation, thereby shaping the magnitude, type, and duration of immune responses (20, 21). Beyond local immune modulation, cytokines also interact with neuroendocrine pathways, including the hypothalamic–pituitary–adrenal axis, contributing to systemic acute-phase responses (22). The liver is the major source of acute-phase proteins (APPs), a group of plasma proteins that play a critical role in the innate immune response by limiting tissue damage, restoring homeostasis, and inhibiting microbial proliferation independently of antibodies (23). During infection, inflammation, or other physiological challenges, the concentrations of APPs in the circulation undergo marked changes, with positive APPs increasing and negative APPs decreasing in a species-specific manner. These alterations provide valuable diagnostic and prognostic indicators of inflammatory and infectious conditions (24, 25). In ruminants, haptoglobin (Hp) is considered the principal acute-phase protein, while serum amyloid A (SAA) is another major positive APP that increases rapidly during the acute-phase response. This response represents a systemic reaction to disturbances in homeostasis caused by infection, tissue injury, trauma, surgery, neoplastic growth, or immune-mediated disorders (26).

Modern molecular genetic tools offer valuable complementary strategies for disease control by improving animal health and resilience (27). Numerous genetic markers, particularly single nucleotide polymorphisms (SNPs), have been identified as key determinants of disease susceptibility or resistance in sheep, highlighting the influence of the host genetic background on disease outcomes (28–30). Despite these advances, the complex interactions among immune responses, antioxidant defense systems, genetic polymorphisms, and changes in gene expression in sheep with mange remain insufficiently understood. Relatively few studies have examined host genetic variation linked to disease susceptibility, despite the fact that molecular investigations of mange in sheep have mostly concentrated on parasite detection and epidemiology (31). According to mounting data from livestock, polymorphisms in immune-regulatory genes affect host resistance to parasitic and infectious illnesses (32). Therefore, identifying SNPs in genes related to oxidative stress and inflammation may help build genetic biomarkers for breeding programs and enhance our knowledge of host vulnerability to mange.

The candidate genes examined in this study were chosen because they reflect interrelated biological pathways known to contribute to host defense against parasite infections (33). Important mediators of innate immune recognition and NF-κB signaling activation after pathogen detection are *CARD9* and *CLEC7A*. While *IL33* acts as an alarmin produced after epithelial injury to boost type-2 immunity during parasite infestation, *TNFAIP3* (A20) is a crucial negative regulator of inflammatory reactions (34, 35). *SLC7A11* controls glutathione synthesis and cystine uptake, preserving intracellular redox homeostasis; *HSPA1A* encodes an inducible molecular chaperone that shields cells from inflammatory damage; *SESN2* and *ATF4* are important regulators of oxidative and endoplasmic reticulum stress responses (36). These genes were chosen to offer an integrated assessment of immunological activation, cellular stress adaptability, and host genetic susceptibility, since mange simultaneously activates inflammatory and oxidative pathways (37).

We hypothesized that natural mange infestation in Barki ewes is associated with distinct alterations in host genetic, transcriptional, immunological, and oxidative stress responses and that polymorphisms in selected immune- and antioxidant-related genes may contribute to individual variation in susceptibility to the disease. Accordingly, the present study aimed to determine the prevalence of mange mites in Barki sheep and investigate single nucleotide polymorphisms (SNPs) and gene expression profiles of selected immune- and oxidative stress-related genes (*CARD9, CLEC7A, TNFAIP3, IL33, SESN2, ATF4, HSPA1A*, and *SLC7A11*), together with serum acute-phase proteins, cytokines, and antioxidant biomarkers. By integrating these molecular and biochemical approaches, this study sought to provide a comprehensive understanding of the host responses associated with naturally occurring ovine mange and identify candidate molecular markers that warrant further investigation in future studies.

## 2 Materials and methods

### 2.1 Animals and study design

This investigation was designed as a cross-sectional survey with a subsequent case–control comparison and was conducted in 2025–2026 on privately owned Barki sheep farms in Dakahlia Governorate, Egypt. A total of 320 adult Barki ewes aged 4–9 years (mean ± SD: 6.1 ± 1.9 years) and weighing 35.5–48.9 kg (mean ± SD: 42.2 ± 6.7 kg) were clinically examined in this study.

Inclusion criteria for enrollment were adult Barki ewes within the specified age range that were maintained under similar management and feeding conditions on the participating farms. Sheep exhibiting clinical signs suggestive of mange, including pruritus, alopecia, crust formation, hyperkeratosis, and skin thickening, were eligible for the diseased group, provided that the diagnosis was confirmed by microscopic examination of deep skin scrapings from the affected area. Clinically healthy ewes with normal rectal temperature, pulse rate, respiratory rate, appetite, and body condition; no dermatological lesions; and no clinical evidence of systemic illness were eligible for the control group.

Exclusion criteria included sheep younger than 4 or older than 9 years, pregnant or lactating ewes, animals with concurrent infectious, parasitic, metabolic, or systemic diseases, those receiving antiparasitic, antimicrobial, anti-inflammatory, or antioxidant treatments before sampling, and animals with incomplete clinical records or inadequate biological samples.

All animals underwent a comprehensive clinical examination, including rectal temperature, pulse rate, and respiratory rate measurements, following standard veterinary procedures (38). Sheep exhibiting clinical signs suggestive of mange underwent parasitological confirmation through microscopic examination of deep skin scrapings. All clinically affected sheep were confirmed to be positive for mange by microscopic identification of mites and/or their developmental stages in deep skin scrapings. Based on the clinical and parasitological findings, 80 sheep with confirmed mange infestation were assigned to the diseased group (DG). The remaining 240 clinically healthy sheep, which showed normal vital parameters, no skin lesions, normal appetite and body condition, and no evidence of mange, were included in the control group (CG).

The diseased sheep exhibited characteristic clinical manifestations of mange, including weight loss, emaciation, intense pruritus, erythema of the affected skin, crust formation, hyperkeratosis, alopecia, skin thickening with a rough and wrinkled appearance, and a dull, rough fleece. Blood samples were subsequently collected from both groups for biochemical, immunological, oxidative stress, and molecular analysis.

All ewes were maintained in semi-covered housing systems and fed a daily ration of concentrate feed and alfalfa hay, with seasonal green fodder provided when it was available. Diets were formulated to meet the maintenance and production requirements of adult Barki ewes, supplying approximately 12–14% crude protein and adequate metabolizable energy. Clean drinking water was provided ad libitum. None of the enrolled ewes had received acaricidal treatment before clinical examination or sample collection. As part of the routine flock health management program, all animals had previously received albendazole (Arab Veterinary Industrial Company [AVICO], Amman, Jordan) orally at 10 mg/kg body weight for the control of gastrointestinal helminths. Because albendazole has no activity against *Sarcoptes* mites, it was not administered as a treatment for sarcoptic mange and, therefore, was not expected to influence the diagnosis or study outcomes.

### 2.2 Blood sampling

Approximately 10 mL of blood was collected from each ewe by jugular venipuncture at the time of clinical examination and confirmation of sarcoptic mange infestation (or routine clinical examination for healthy controls) before the administration of any antiparasitic or supportive treatment. Because this was a cross-sectional study involving naturally infested animals, the exact time of disease onset could not be determined. Each blood sample was divided into three aliquots of equal volume. The first aliquot was collected in EDTA-containing tubes and processed immediately for total RNA extraction and real-time PCR analysis. The second aliquot was collected in tubes containing 5000 IU heparin calcium as an anticoagulant for plasma separation. The third aliquot was transferred into plain tubes without an anticoagulant and allowed to clot at room temperature for 20–30 min before centrifugation at 3,000 rpm for 20 min to obtain serum. All samples were immediately placed on crushed ice and transported to the laboratory for processing. Plasma was separated by centrifugation at 3,000 rpm for 20 min. The obtained serum and plasma samples were stored at −20 °C until immunological and antioxidant analyses were performed.

### 2.3 Parasitological examination and skin scraping

Only sheep exhibiting clinical signs suggestive of mange underwent parasitological examination to confirm the diagnosis. Deep skin scrapings were collected from the periphery and margins of active lesions at various body sites, including the head, face, neck, ears, tail, and trunk. Scrapings (approximately 1–2.5 cm^2^) were obtained from affected areas and placed in black plastic containers following the method described by (39). All samples were processed within 12 h of collection. Briefly, 20 mL of 10% potassium hydroxide (KOH) solution was added to each sample to digest tissue material, followed by boiling for 5–10 min. The digested samples were then centrifuged at 1,500 rpm for 4–5 min. The resulting supernatant and sediment were examined microscopically on glass slides, and permanent mite mounts were prepared as described by (40). Mite identification was performed based on morphological characteristics according to (41).

### 2.4 Genetic polymorphisms and gene expression analysis of immune and antioxidant markers

Total RNA was isolated from whole blood samples collected from healthy and mange-infected sheep using TRIzol reagent (Invitrogen, Thermo Fisher Scientific, USA), followed by purification using the RNeasy Mini Kit (Qiagen, Hilden, Germany; Cat. No. 74104) according to the manufacturer’s protocol.

The concentration and purity of the extracted RNA were evaluated using a NanoDrop ND-1000 spectrophotometer (Thermo Fisher Scientific, USA). Only RNA samples with acceptable purity ratios (A260/A280 between 1.8 and 2.0) were used for downstream applications.

Complementary DNA (cDNA) was synthesized from purified RNA using a reverse transcription kit (Thermo Fisher Scientific, Cat. No. EP0441) according to the supplier’s instructions. The transcriptional profiles of selected immune-related genes (*CARD9, CLEC7A, TNFAIP3,* and *IL33*) and oxidative stress and cellular defense genes (*SESN2, ATF4, HSPA1A*, and *SLC7A11*) were quantified using quantitative real-time reverse transcription PCR (qRT-PCR). Amplification reactions were performed using SYBR Green chemistry (2× SensiFAST SYBR Mix, Bioline, Cat. No. BIO-98002); alternatively, it was confirmed using the QuantiTect SYBR Green PCR Kit (Qiagen, Cat. No. 204141).

Gene-specific primers were designed based on *Ovis aries* gene sequences retrieved from the GenBank database of the National Center for Biotechnology Information (NCBI) (Table 1). Conserved regions were identified through multiple sequence alignment using ClustalW software to ensure cross-sample amplification efficiency. Primer candidates were generated using Primer-BLAST (NCBI) and assessed for specificity, melting temperature, GC content, and the absence of secondary structures or primer-dimer formation. The glyceraldehyde-3-phosphate dehydrogenase (*GAPDH*) gene was used as an internal reference gene to normalize the expression levels.

**Table 1:** Oligonucleotide primer sequences, accession numbers, annealing temperatures and PCR product sizes of immune and antioxidant genes used in real-time PCR.

Examined marker	Primer	Product size (bp)	Annealing temperature (°C)	GenBank isolate
*CARD9*	F5′-ATGTCCGACTACGAGAACGAGG-3 R5′-ACCTCGCTCATCAGCAGCTGCGT-3′	368	55	XM_027966185.3
*CLEC7A*	F5′- GATGGATATACTCAATTAGACT -3′ R5′-CCACTGAGCCACCAGGGGAATC-3′	489	58	XM_060413428.1
*TNFAIP3*	F5′-AGGCGGCAAAAGAACCAAACGA-3′ R5′-AGGCTCTGGGCCAGCTGTGAGG-3′	479	58	XM_060419284.1
*IL33*	F5′-ATGAAGCCTAAAATGAAGTAT-3′ R5′-CACCTTGTCTTTGATACTTGAT-3′	369	58	XM_060409448.1
*SESN2*	F5′-′ ATGATCGTTGCGGACTCCGAGT-3′ R5′-GTGGAGGCCAAGCAGCCACTCAG-3′	423	55	XM_027965423.2
*ATF4*	F5′-ATCGCTACCCTGGCGGTTAAG-3′ R5′GTCATCATAGAGGCTGCCACTCA-3′	459	58	XM_060411578.1
*HSPA1A*	F5′-CCGGTGGTGCAGTCGGACATGA-3′ R5′-CACCTCGAAGATGCCGTCGTCG-3′	417	55	NM_001267874.1
*SLC7A11*	F5′-ATTATGGAATGTATGCGTACGC-3′ R5′-AGCTGGCAGAGGAGTGTGCTTC-3′	387	55	NM_001252182.1
*GAPDH*	F5′-TGGTGAAGGTCGGAGTGAAC-3′ R5′-CCGTTCTCTGCCTTGACTGT-3′	187	58	NM_001190390.1

CARD9, Caspase Recruitment Domain Family Member 9; CLEC7A, C-type Lectin Domain Containing 7A; TNFAIP3, Tumor Necrosis Factor Alpha–Induced Protein 3; IL33, Interleukin-33; SESN2, Sestrin-2; ATF4, Activating Transcription Factor 4; HSPA1A, Heat Shock Protein Family A Member 1A; SLC7A11, Solute Carrier Family 7 Member 11. GAPDH, glyceraldehyde-3-phosphate dehydrogenase.

Gene expression analysis was performed using RNA extracted from whole blood collected from all healthy (*n* = 240) and mite-infected sheep (*n* = 80), with each animal representing one biological replicate. Each sample was analyzed in triplicate, and the mean Ct value was used for statistical analysis. Each qRT-PCR reaction was performed in a final volume of 25 μL containing 3 μL of total RNA template, 4 μL of 5× TransAmp buffer, 0.25 μL reverse transcriptase enzymes, 0.5 μL of each forward and reverse primer, 12.5 μL of 2× SYBR Green PCR Master Mix, and 4.5 μL RNase-free water.

The thermal cycling conditions included reverse transcription at 50 °C for 30 min, initial denaturation at 94 °C for 10 min, followed by 40 amplification cycles consisting of denaturation at 94 °C for 15 s, gene-specific annealing for 60 s (temperatures listed in Table 1), and extension at 72 °C for 30 s. Melting curve analysis was conducted at the end of each run to verify amplification specificity and exclude nonspecific products.

Relative gene expression levels were calculated using the comparative Ct (2^−ΔΔCt) method, with *GAPDH* as the endogenous control (42). Expression profiles were compared between healthy and mite-infected sheep to evaluate alterations in immune signaling pathways and oxidative stress responses associated with parasitic infestation.

### 2.5 Purification, sequencing, and SNP analysis of qRT-PCR amplicons

Prior to sequencing, amplification artifacts, including primer dimers, nonspecific products, and residual reaction contaminants, were removed to ensure high-quality DNA templates. Real-time PCR products displaying the expected fragment size were purified using a commercial PCR purification kit (Jena Bioscience, Germany; Cat. No. PP-201×s) according to the manufacturer’s instructions, following the principles described for nucleic acid purification protocols (43).

The concentration and purity of the purified amplicons were evaluated using a NanoDrop UV–Vis spectrophotometer (Q5000, USA) to confirm the adequate DNA quantity and optimal absorbance ratios required for sequencing analysis. Purified qRT-PCR products obtained from both healthy and scab-infected sheep were subsequently subjected to bidirectional sequencing using forward and reverse primers on an ABI 3730XL automated DNA sequencer (Applied Biosystems, USA). Sequencing reactions were performed according to the Sanger chain termination method (44).

Raw sequencing chromatograms were visually inspected and edited using Chromas software version 1.45, and sequence identity was verified using the Basic Local Alignment Search Tool (BLAST) available through the National Center for Biotechnology Information (NCBI) database (45). Each obtained sequence was aligned with the corresponding *Ovis aries* reference sequence retrieved from GenBank to detect nucleotide variations. The identified sequence differences were recorded and classified as single nucleotide polymorphisms (SNPs).

To assess the potential functional consequences of the detected polymorphisms, nucleotide sequences were translated into predicted amino acid sequences and comparatively analyzed using MEGA version 6 software. Multiple sequence alignment and comparative analyses were conducted among samples from healthy and mange-infected sheep to evaluate genetic variability within the investigated immune and oxidative stress–related genes (46).

### 2.6 APPs, immunological, and antioxidants analysis

The following APPs, immunological, and antioxidant parameters were measured using the collected serum samples according to the manufacturers’ protocols:

Acute phase proteins: Serum haptoglobin (Hp) was measured using Eagle Biosciences (Columbia) ELISA kits, serum amyloid A (SAA) and plasma fibrinogen (Fb) concentrations were measured using IBL International Crop (Canada) ELISA kits, and serum caeruloplasmin (Cp) levels were measured using Arbor Assays DetectX (USA) kits.

Cytokines: Pro-inflammatory cytokines (IL-1*α*, IL-1β, IL-6, IFN-*γ*, TNF-α, pg/mL) and the anti-inflammatory cytokine IL-10 (pg/mL) were quantified using ELISA kits from MyBiosecure.

Oxidative stress markers and antioxidants: Catalase (CAT, U/L), superoxide dismutase (SOD, U/mL), glutathione peroxidase (GPx, U/mL), glutathione (GSH, mg/dL), and malondialdehyde (MDA, nmol/mL) were measured spectrophotometrically using commercial kits from Biodiagnostic Company.

### 2.7 Statistical analysis

Statistical analyses were performed using SPSS version 20 (Chicago, Armonk, NY, USA). Differences in APPs, immunological and antioxidant parameters, and gene expression between healthy and diseased ewes were assessed using an independent-samples t-test because only two experimental groups were included in the study. Relative gene expression was calculated using the 2^−ΔΔCt method, and statistical analyses were performed on ΔCt values. Fold changes (infected versus healthy) were calculated from the relative expression values, and the corresponding 95% confidence intervals (95% CIs) were determined to assess the precision of the estimated fold changes. Gene expression data are presented as box-and-whisker plots with individual data points, where the box represents the interquartile range (IQR), the center line indicates the median, and the whiskers represent the minimum and maximum values of the data. Statistical significance was set at *p* < 0.05.

Chi-square tests were used to evaluate differences in the frequencies of gene SNPs between groups. Additionally, linear discriminant analysis (LDA) was conducted to assess whether the average SNP scores for the eight genes could distinguish between healthy and diseased animals, with the gene averages as predictor variables and health status (diseased vs. healthy) as the grouping variable. To evaluate the relationship between gene expression and serum profiles of immunological and antioxidant markers in diseased animals, Pearson’s correlation was used. Consideration was given to The P-value and correlation coefficient (r) was considered. Statistical significance was set at *p* < 0.05.

## 3 Results

### 3.1 Clinical findings

**Figure 1:**
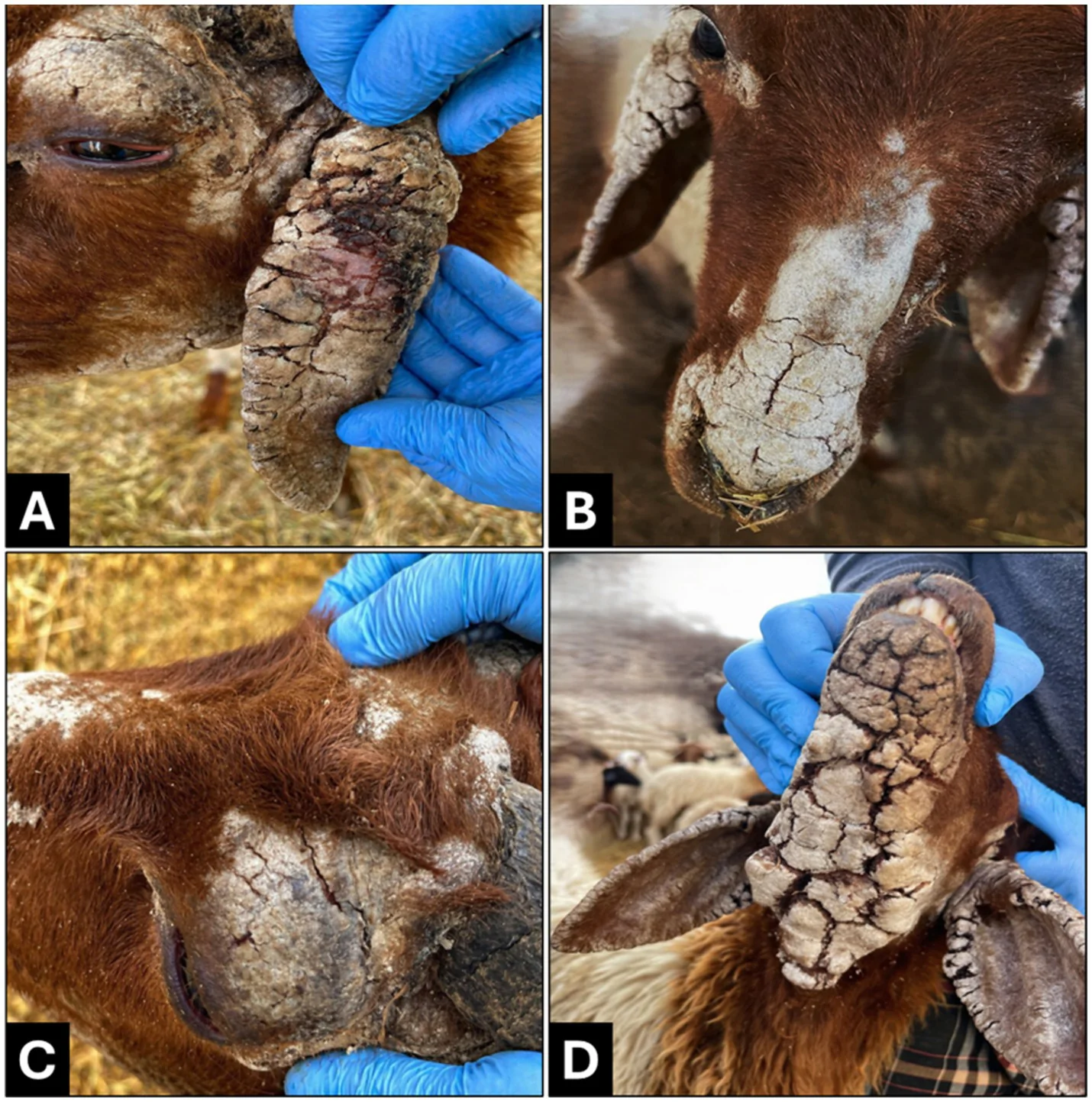
Gross clinical lesions associated with mange in sheep **(A)** Clinical appearance of mange in sheep showing thickened, crusted, and fissured lesions on the ear; **(B)** Crusted mange lesions affecting the muzzle and nasal region of an infected sheep; **(C)** Extensive hyperkeratotic and crusted mange lesions involving the facial and periocular regions of a sheep; **(D)** Severe crusted facial and auricular lesions in mange-infested sheep.

Of the 320 sheep examined, 80 (25%) were diagnosed with mange. Clinically, the diseased group (DG) exhibited significantly higher pulse and respiratory rates (*p* < 0.05) than the control group (CG), with mean values of 101.1 ± 0.35 beats/min and 47.2 ± 0.5 breaths/min in the DG versus 75 ± 10 beats/min and 25 ± 5 breaths/min in the CG, respectively. No significant differences were observed in the body temperatures between the two groups. In addition, the diseased ewes had a significantly lower body condition score (BCS) than the control group (*p* < 0.05), indicating a progressive loss of body reserves associated with chronic mange infestation. Affected ewes exhibited clinical signs characteristic of *sarcoptic* mange, including severe pruritus, dermatitis, alopecia, emaciated body condition, weakness, anorexia, depression, anemia (pale mucous membranes), and prominent bony projections. Skin lesions were distributed across multiple body regions, including the head, neck, ears, periocular area, nose, muzzle, and mandible (Figure 1). The affected skin appeared rough, corrugated, and covered with grayish, chalky scabs, which often cracked into deep fissures exuding sero-hemorrhagic exudates.

### 3.2 Parasitological findings

**Figure 2:**
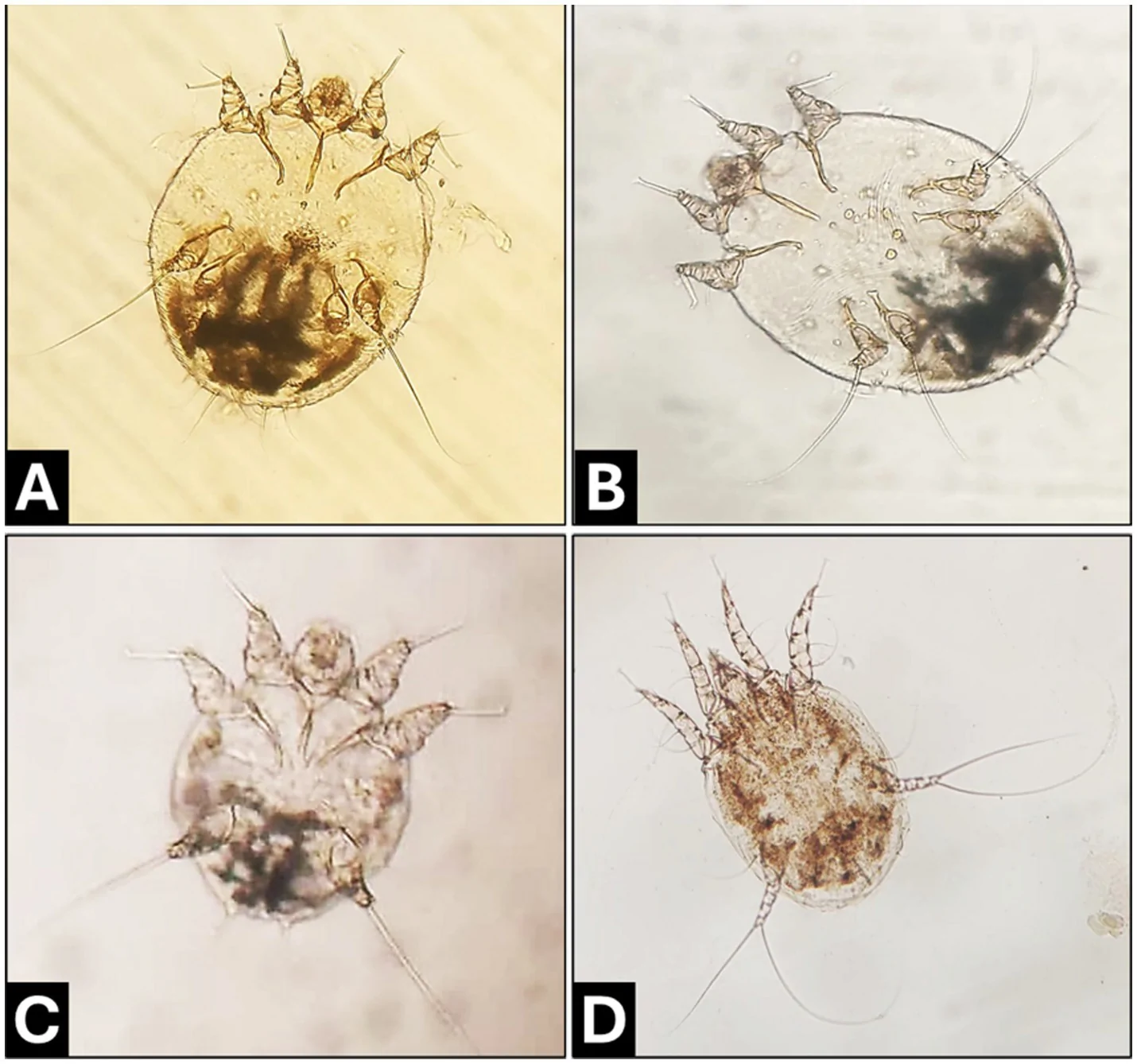
Photomicrograph (40×) of the ventral view of a mange mite obtained from skin scrapings, used for morphological identification of mange infestations in livestock **(A)** Male *Sarcoptes scabiei* with oval, flattened body and prominent anterior legs; **(B)** Female *Sarcoptes scabiei* mite with rounded body and short legs not extending beyond the margin; **(C)** Larval stage of *Sarcoptes scabiei* with small rounded body and three pairs of legs; **(D)** The *Psoroptes ovis* larva has an oval, dorsoventrally flattened, semi-transparent body with three pairs of anterior legs bearing setae and pedicels ending in suckers for attachment.

Microscopic examination of skin scrapings confirmed mange mites in all clinically affected animals (25%). Among the 320 ewes, 70 cases (21.9%) were single infestations, while 10 cases (3.1%) showed mixed infestations. *Sarcoptes* spp. were the predominant mites, detected in 60 of 80 affected animals (75%) and 18.75% of the total population, whereas *Psoroptes* spp. were found in 20 of 80 affected animals (25%) and 6.25% of the total examined, as summarized in Figure 2 and Table 2.

**Table 2:** Prevalence and distribution of mange mites in examined ewes.

Category	Number of animals	% of Total examined (*n* = 320)	% of Infected animals (*n* = 80)
Apparently healthy	240	75.0	––
Total infected	80	25.0	100
Single infection	70	21.9	87.5
Mixed infection (Sarcoptes + Psoroptes)	10	3.1	12.5
*Sarcoptes* spp.	60	18.75	75
*Psoroptes* spp	20	6.25	25

### 3.3 Expression patterns of immune and antioxidant genes

**Figure 3:**
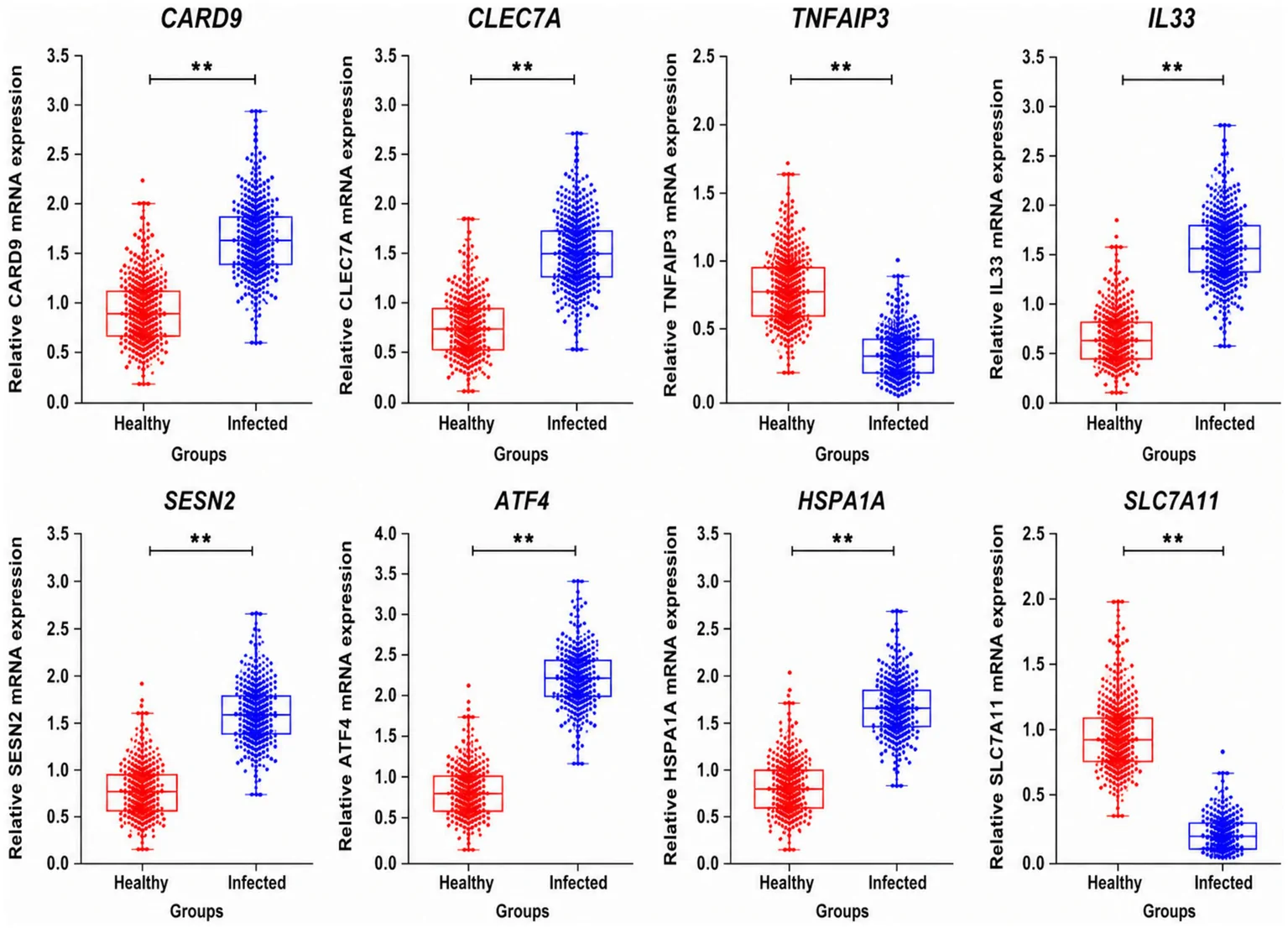
Relative mRNA expression levels of CARD, CLEC7A, TNFAIP3, IL33, SESN2, ATF4, HSPA1A, and SLC7A11 in healthy (*n* = 240) and infected (*n* = 80) ewes. Relative expression levels were calculated using the 2^^−ΔΔCt^ method. Individual data points are overlaid on box-and-whisker plots, where the box represents the interquartile range (IQR), the central line indicates the median, and the whiskers extend to the minimum and maximum values. Fold-change estimates with their corresponding 95% confidence intervals (95% CIs) are reported in the Results section. Statistical significance between groups was assessed using an independent-samples *t*-test based on Δ*Ct* values. **p < 0.01 was considered statistically significant.

As illustrated in Figure 3, distinct differences in the transcriptional activity of the investigated immune- and oxidative stress-related genes were observed between healthy sheep and animals affected by mange infestation. The infected group exhibited significantly higher expression levels of *CARD9, CLEC7A, IL33, SESN2, ATF4,* and *HSPA1A* than the healthy controls (*p* < 0.05), whereas *TNFAIP3* and *SLC7A11* expression was significantly downregulated in the infected group. The estimated fold changes (infected versus healthy) were 1.82 (95% CI: 1.72–1.94) for *CARD9*, 1.94 (95% CI: 1.80–2.10) for *CLEC7A*, 0.56 (95% CI: 0.52–0.61) for *TNFAIP3*, 2.17 (95% CI: 2.01–2.34) for IL33, 1.83 (95% CI: 1.70–1.96) for *SESN2,* 2.01 (95% CI: 1.89–2.14) for *ATF4*, 1.79 (95% CI: 1.70–1.90) for *HSPA1A*, and 0.43 (95% CI: 0.40–0.47) for *SLC7A11.*

Among the healthy sheep, *ATF4* exhibited the highest baseline expression (mean relative expression = 1.125), whereas *IL33* exhibited the lowest baseline expression (mean relative expression = 0.875). In contrast, among the infected sheep, *IL33* displayed the greatest increase in expression (2.17-fold), whereas *SLC7A11* exhibited the greatest reduction (0.43-fold). Collectively, these findings demonstrate marked alterations in immune signaling, oxidative stress regulation, and cellular stress response pathways associated with natural mange infestation.

### 3.4 Genetic variability and molecular polymorphism analysis of immune and antioxidant genes

Sequence analysis of the PCR-derived amplicons revealed multiple genetic polymorphisms associated with the mange status in sheep. Amplified fragments corresponding to *CARD9* (368 bp), *CLEC7A* (489 bp), *TNFAIP3* (479 bp), *IL33* (369 bp), *SESN2* (423 bp), *ATF4* (459 bp), *HSPA1A* (417 bp), and *SLC7A11* (387 bp) were successfully sequenced and compared between healthy and infected animals. Comparative alignments of the investigated genes between healthy and mange-infected Barki sheep, relative to the corresponding reference sequences retrieved from GenBank, are presented in Supplementary Figures S1–S8.

Alignment of the coding sequences demonstrated the presence of exon-based nucleotide variations across all investigated genes (Table 3). A total of 14 single nucleotide polymorphisms (SNPs) were identified, including six non-synonymous substitutions leading to amino acid changes and eight synonymous substitutions without alterations in the protein sequence.

**Table 3:** Distribution of immune and antioxidant genetic markers and single nucleotide variations in healthy and mange-affected sheep.

Gene	SNPs	Healthy	Mange	Total	Chi square value *X*^2^	*p*-value	Kind of inherited change	Amino acid order and sort
		*n* = 240	*n* = 80	*n* = 320				
*CARD9*	A37G	181/240	-/80	181/320	119	0.001	Non-synonymous	13 T to A
	T303C	165/240	-/80	165/320	98.6	0.001	Synonymous	101 R
*CLEC7A*	G456A	173/240	-/80	173/320	108.3	0.001	Synonymous	152 A
*TNFAIP3*	C110T	-/240	49/80	49/320	186	0.001	Non-synonymous	37 A to V
*IL33*	G175T	146/240	-/80	146/320	78.7	0.001	Non-synonymous	59 V to F
*SESN2*	A99G	-/240	68/80	68/320	276.2	0.001	Synonymous	33 R
	A125G	-/240	38/80	38/320	139	0.001	Non-synonymous	42 E to G
	T333C	152/240	-/80	152/320	84.5	0.001	Synonymous	111 A
*ATF4*	C102A	-/240	57/80	57/320	222.5	0.001	Synonymous	34 P
	A221G	-/240	42/80	42/320	155.7	0.001	Non-synonymous	74 Q to R
*HSPA1A*	C316G	-/240	73/80	73/320	302.1	0.001	Non-synonymous	106 H to D
*SLC7A11*	C111T	-/240	67/80	67/320	271.1	0.001	Synonymous	37 V
	C270T	138/240	-/80	138/320	71.4	0.001	Synonymous	90 G
	C312T	-/240	34/80	34/320	122.7	0.001	Synonymous	104 Y

CARD9, Caspase Recruitment Domain Family Member 9; CLEC7A, C-type Lectin Domain Containing 7A; TNFAIP3, Tumor Necrosis Factor Alpha–Induced Protein 3; IL33, Interleukin-33; SESN2, Sestrin-2; ATF4, Activating Transcription Factor 4; HSPA1A, Heat Shock Protein Family A Member 1A; SLC7A11, Solute Carrier Family 7 Member 11.

Sequence alignment of the amplified coding regions identified 14 SNPs distributed across the eight investigated genes. These variants consisted of six non-synonymous substitutions causing amino acid changes and eight synonymous substitutions that did not alter the encoded proteins. The detected polymorphisms included transitions (AG and CT) and transversions (AT, AC, GT, and CG). Several SNPs were uniquely detected in either healthy or mange-infected sheep, whereas others were shared between both groups but differed significantly in their allele frequency. Non-synonymous variants were mainly identified within *TNFAIP3, IL33, ATF4, HSPA1A* and *SLC7A11*. Several polymorphisms showed clear group-specific distribution patterns in the present study. Variants in *CARD9, CLEC7A, IL33*, and selected loci of *SLC7A11* were predominantly detected in healthy sheep, whereas mutations in *TNFAIP3, SESN2, ATF4*, *HSPA1A*, and additional *SLC7A11* loci were mainly observed in mange-infected animals.

Statistical evaluation revealed highly significant differences in SNP frequencies between the healthy and infected groups (*p* < 0.005). Chi-square analysis further confirmed that the distribution of detected polymorphisms across all examined genes differed significantly according to disease status (*p* < 0.05), supporting an association between genetic variation and susceptibility to mange infestation (Table 3).

### 3.5 APPs, antioxidant and immunological profile

Evaluation of acute-phase proteins (APPs) demonstrated significant increases (*p* < 0.05) in the serum concentrations of haptoglobin, serum amyloid A and ceruloplasmin (3.7 ± 0.1 g/dL, 8.2 ± 0.2 mg/L and 5.8 ± 0.1 mg/dL), respectively and plasma fibrinogen (291.6 ± 10.1 mg/dL) in diseased ewes compared with healthy controls. Furthermore, serum levels of the pro-inflammatory cytokines IL-1*α* (145.6 ± 0.1 pg/mL), IL-1β (152.3 ± 1.5 pg/mL), IL-6 (114.6 ± 0.2 pg/mL), and TNF-α (166.9 ± 0.7 pg/mL) were significantly elevated (*p* < 0.05) in infected animals, indicating activation of the innate immune response. Conversely, the anti-inflammatory cytokine IL-10 was significantly reduced (33.5 ± 0.5 pg/mL) in infected ewes relative to healthy controls. Assessment of oxidative stress biomarkers revealed significant decreases in catalase (CAT), superoxide dismutase (SOD), glutathione (GSH), and glutathione peroxidase (GPx), accompanied by a marked increase in malondialdehyde (MDA) levels, reflecting enhanced oxidative stress and compromised antioxidant defense mechanisms in infected ewes (Table 4).

**Table 4:** APPs, immunological profile and antioxidant/oxidative stress markers of healthy (*n* = 150) and diseased (*n* = 50) Barki ewes.

Parameters	Healthy ewes	Diseased ewes	*p*-values
Haptoglobin (g/dL)	0.24 ± 0.01	3.7 ± 0.1*	0.001
Serum amyloid A (mg/L)	2.8 ± 0.1	8.2 ± 0.2*	0.001
Fibrinogen (mg/dL)	141.6 ± 4.4	291.6 ± 10.1*	0.001
Ceruloplasmin (mg/dL)	3.4 ± 0.1	5.8 ± 0.1*	0.001
IL1α (pg/mL)	39.4 ± 0.6	145.6 ± 0.1*	0.001
IL 1β (pg/mL)	37 ± 1.6	152.3 ± 1.5*	0.008
IL 6 (pg/mL)	25.5 ± 1.3	114.6 ± 0.2*	0.005
IL10 (pg/mL)	99.5 ± 0.6	33.5 ± 0.5*	0.002
TNFα (pg/mL)	29.5 ± 0.4	137.4 ± 0.5*	0.001
SOD (U/mL)	46.3±0.8	30±0.5*	0.001
CAT (U/L)	29±0.5	20±0.8*	0.001
GSH(mg/dL)	41±1.5	27.6±1.4*	0.003
GPx (U/mL)	42.3±2.8	32±0.5*	0.002
MDA (nmol/mL)	12.8±0.9	16.9±1.1*	0.01

IL-1α: Interleukin 1 alpha; IL-1β: Interleukin 1 beta; IL-6: Interleukin 6; TNF- α: Tumor necrosis factor- alpha; IL10: Interleukin 10; SOD: Superoxide dismutase; CAT: Catalase; GSH: Glutathione reduced; GPx: Glutathione peroxidase; NO: Nitric oxide; MDA: Malondialdhyde. The symbol * denotes significance when *p* < 0.05.

### 3.6 Correlation between gene expression pattern and serum profile of immunological markers in diseased sheep

**Figure 4:**
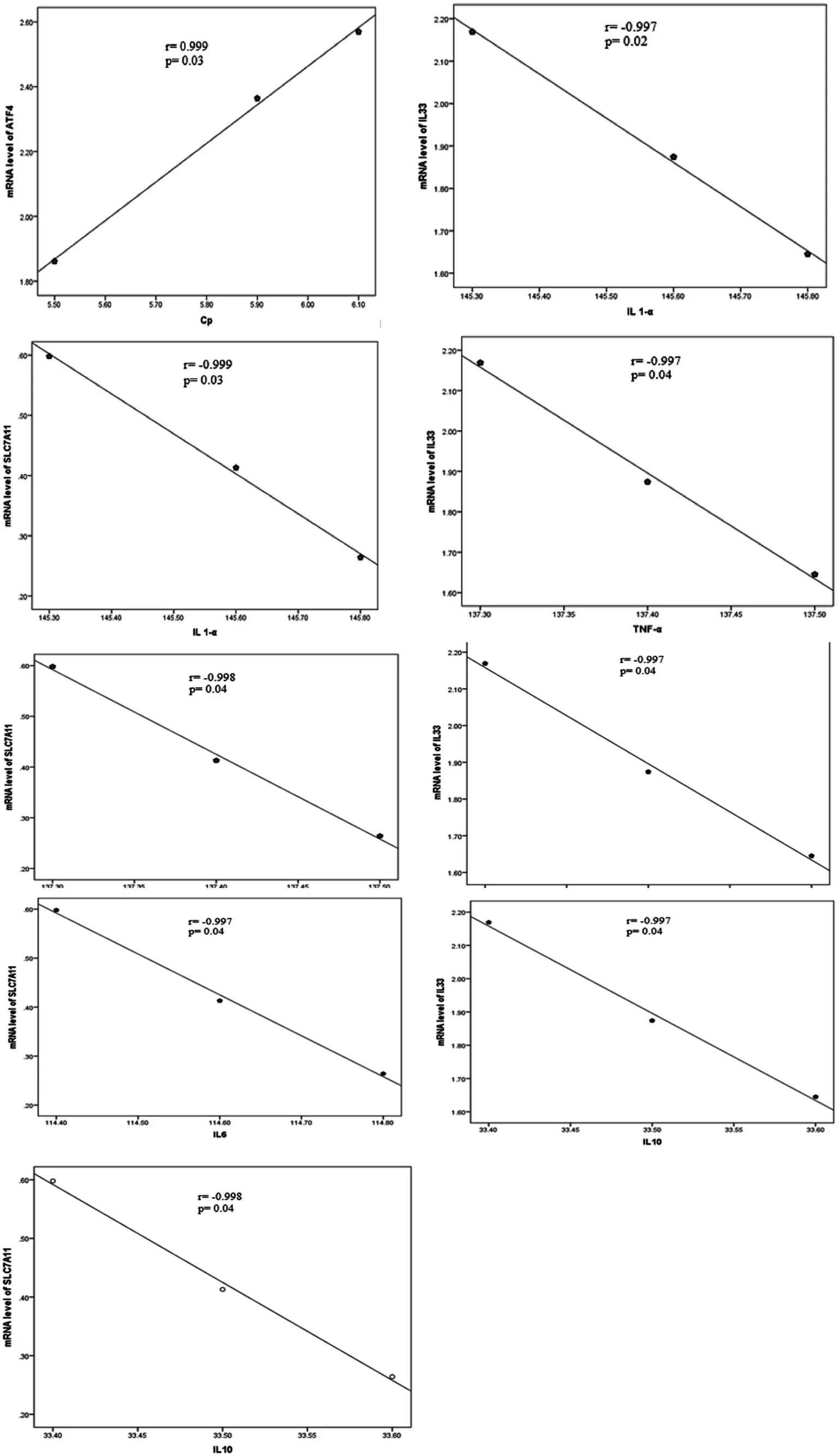
The correlation between the genetic parameters and immunological parameters in diseased sheep. Pearson’s correlation test considered significant when *p* < 0.05.

Figure 4 illustrates the association between gene expression profiles and serum immunological markers in sheep. Because the correlation analysis was conducted on a relatively small subset of diseased animals, the correlation coefficients should be interpreted cautiously, as the limited sample size can inflate Pearson's correlation estimates. Therefore, these findings are considered exploratory and warrant validation in larger, independent populations. Within these limitations, a significant positive correlation was observed between *ATF4* mRNA expression and serum ceruloplasmin (Cp) concentration (*r* = 0.999, *p* = 0.03). In contrast, *IL33* mRNA expression was significantly negatively correlated with serum IL-6, IL-10, and TNF-*α* levels (*r* = −0.997, *p* = 0.04) and IL-1α (*r* = −0.999, *p* = 0.02). Similarly, *SLC7A11* mRNA expression was negatively correlated with serum IL-6, IL-10, and TNF-α concentrations (*r* = −0.998, *p* = 0.04) and IL-1α (*r* = −0.999, *p* = 0.03).

## 4 Discussion

Mange is a highly contagious and debilitating skin disease in sheep that significantly compromises their health and productivity. Consistent with previous studies (4, 8–10, 47, 48), the affected ewes in this study displayed tachycardia and tachypnea, along with dermatitis characterized by severe pruritus and self-inflicted skin lesions, likely resulting from toxins secreted by the mites and persistent scratching of the skin.

The present study revealed an overall mange prevalence of 25% among the examined sheep, indicating a considerable parasitic burden under field conditions. This finding is consistent with several studies in Egypt, where prevalence rates vary according to management practices, environmental factors, and diagnostic approaches. For instance, Mazyad et al. (49) reported a prevalence of 20.98% in sheep from North Sinai, while (4) recorded a prevalence of 24.5% in the Menoufia Governorate. In contrast, studies conducted in Giza Governorate reported a markedly higher infestation rate (83.2%) of mange mites in Egyptian buffaloes, whereas lower rates have been documented in other regions, reflecting notable geographical variations. These findings confirm that *Sarcoptes scabiei* remains a significant health concern in sheep, underscoring the need for better management, regular screening, and effective control. Sustainable control of ectoparasitic diseases should integrate routine surveillance, strategic use of acaricides, improved farm hygiene, and resistance management to reduce parasite transmission and maintain treatment efficacy, which is consistent with current recommendations for integrated ectoparasite control in livestock (50).

Microscopic examination confirmed infestation in all clinically affected animals (25%), supporting its use as a rapid diagnostic tool. However, it may miss early or subclinical cases, highlighting the need for molecular testing methods. Recent investigations have demonstrated that molecular techniques provide valuable tools for accurate ectoparasite identification, assessment of ectoparasite biodiversity, and detection of associated pathogens, thereby improving epidemiological surveillance and control programs (51, 52). Single infections (21.9%) predominated over mixed infections (3.1%), consistent with reports that mange is typically caused by a single species, although mixed infestations can occur under poor management and high stocking densities (53). The predominance of *Sarcoptes scabiei* in our study aligns with recent reports showing *sarcoptic* mange is the most common mite infestation in sheep (54). Globally, it spreads rapidly in overcrowded or unhygienic herds due to its burrowing behavior and direct life cycle (55), whereas *Psoroptes ovis* causes superficial infestations influenced mainly by environmental conditions. The higher prevalence observed in this study may reflect local risk factors in Egypt, such as limited parasite control, scarce acaricide use, close animal contact, and seasonal and climatic influences on mite survival. These findings confirm that *Sarcoptes scabiei* remains a significant health concern in sheep, underscoring the need for better management, regular screening, and effective control measures (56).

In the present study, combined transcriptional and genetic analyses revealed substantial differences between healthy and mite-infected sheep in both immune regulatory genes and cellular defense pathways. These findings suggest that disease outcomes are influenced not only by pathogen exposure but also by host molecular responses and underlying genetic variation, as immune activation during mite infestation induces inflammatory mediators and reactive oxygen species that disrupt the oxidant–antioxidant balance and contribute to tissue damage and disease progression (57).

The elevated transcription of *CARD9* in infected sheep indicates the activation of innate immune signaling during mite infestation. *CARD9* functions as an intracellular adaptor that links pattern recognition receptors to downstream inflammatory pathways, particularly NF-κB–mediated cytokine production (58). Increased *CARD9* expression likely reflects the recognition of parasite-associated molecular patterns released during mite invasion of the epidermis. Previous studies investigating ectoparasitic and fungal infections have shown that *CARD9* signaling enhances the recruitment of immune cells to the affected tissues, promoting pathogen clearance (59). However, excessive activation may intensify inflammatory damage, which is a hallmark of mange lesions.

Similarly, *CLEC7A*, a C-type lectin receptor involved in pathogen detection, showed increased expression in infected animals. *CLEC7A* recognizes carbohydrate structures present on microbial and parasitic surfaces and contributes to activation of innate immune responses (60). Enhanced *CLEC7A* transcription observed in diseased sheep may therefore represent a compensatory defense mechanism attempting to limit parasite proliferation. Comparable activation of lectin-mediated pathways has been described in skin parasitic infections where local inflammation is required for parasite control but may simultaneously aggravate tissue injury (59).

In contrast to the upregulated immune activation genes, *TNFAIP3* expression was significantly reduced in the mange-infected sheep. *TNFAIP3* encodes A20, an essential inhibitor of NF-κB signaling that terminates inflammatory responses and prevents immune overactivation (61). Reduced expression suggests impaired anti-inflammatory regulation, which may allow persistent inflammation and contribute to the severity of lesions. The identification of infection-associated polymorphisms within *TNFAIP3* further supports the possibility that genetic variation influences regulatory efficiency, thereby affecting susceptibility to mange.

The increased transcription of *IL33* in infected sheep is consistent with its function as an epithelial-derived danger signal released following tissue damage. *IL-33* promotes type-2 immune responses typically associated with parasitic infestations and allergic inflammation (62). Mite burrowing and epidermal disruption likely stimulate *IL-33* expression, enhancing recruitment of eosinophils and inflammatory leukocytes. Similar *IL-33* activation has been documented in scabies and related mite infestations, where it amplifies cutaneous immune responses (63).

Parasitic skin infections are frequently accompanied by oxidative stress, resulting from inflammatory cell activity and reactive oxygen species production. The significant upregulation of *SESN2, ATF4*, and *HSPA1A* in infected sheep supports the activation of stress-adaptation mechanisms.

*SESN2* is an oxidative stress–responsive protein that regulates cellular redox balance and metabolic adaptation (63). Increased *SESN2* expression may represent a protective response aimed at limiting oxidative damage caused by persistent inflammation.

Among all the genes examined, *ATF4* exhibited the highest transcriptional increase, indicating strong activation of the integrated stress response pathways. *ATF4* regulates genes involved in antioxidant defense, amino acid metabolism, and cellular survival under stress conditions (64). Its marked elevation suggests that mange infestation imposes substantial cellular stress, requiring metabolic reprogramming.

The upregulation of *HSPA1A*, which encodes heat shock protein 70, further confirms the activation of cytoprotective pathways. Heat shock proteins stabilize damaged proteins and assist in cellular recovery during inflammatory and environmental stress (65). Increased *HSPA1A* expression has been observed in several parasitic and dermatological conditions, reflecting attempts to preserve the cellular integrity.

Conversely, *SLC7A11* expression was significantly decreased in the infected sheep. This gene encodes a cystine transporter essential for glutathione synthesis, which is a major intracellular antioxidant molecule (66). Reduced expression may impair the antioxidant capacity, leading to increased oxidative injury and enhanced disease susceptibility. Infection-associated SNPs detected in *SLC7A11* may further compromise transporter function and exacerbate redox imbalance

Gene expression variability may be influenced by other biological processes, even if the reported transcriptional changes are closely linked to mange infestation (67). Transcriptional responses may be influenced by variations in parasite burden, length of infection, severity of skin lesions, nutritional conditions, environmental stress, and individual genetic background (68). Moreover, variations in whole-blood gene expression may be partially explained by alterations in the circulating leukocyte populations during systemic inflammation. To differentiate causative molecular pathways from secondary reactions to infection, longitudinal studies and functional validation are necessary (69).

The identification of 14 SNPs across immune and antioxidant genes demonstrated notable genetic diversity between healthy and infected animals. Non-synonymous substitutions identified in genes such as *TNFAIP3, IL33, ATF4*, and *HSPA1A* may alter protein function and influence signaling efficiency of these proteins. The differences in SNP distribution between groups suggest that specific alleles may contribute to resistance or increased vulnerability to mange infestation.

Previous livestock studies have demonstrated that polymorphisms within immune regulatory genes can influence host responses to parasitic infections by modifying cytokine signaling and oxidative defense capacity (70). The present findings support a similar genetic contribution to sheep mange susceptibility

The oxidative stress profile observed in mange-infested ewes is consistent with an imbalance between reactive oxygen species production and antioxidant defense systems during chronic parasitic skin diseases. Rather than representing an isolated biochemical alteration, the concurrent increase in lipid peroxidation and reduction in antioxidant enzyme activity suggests that oxidative damage may contribute to the tissue injury associated with *sarcoptic* mange. Similar oxidative alterations have been documented in sheep and other animal species affected by ectoparasitic infestations, supporting the concept that oxidative stress is a common component of host responses to persistent mite infections (10, 48, 71–74). Activated neutrophils and macrophages recruited to skin lesions are recognized as sources of reactive oxygen species during parasite-induced inflammation, although the relative contribution of these cells was not directly assessed in the present study. Therefore, the observed oxidative imbalance should be interpreted as consistent with, rather than proving, enhanced inflammatory oxidative activity. Such oxidative injury may also contribute to systemic alterations through lipid peroxidation and cellular membrane damage, potentially exacerbating the tissue dysfunction. This interpretation is compatible with the concurrent hematological and biochemical abnormalities observed in diseased animals but require confirmation by studies specifically evaluating oxidative pathways in mange (72, 75).

The inflammatory biomarker profile further indicated the activation of the innate immune response during natural mange infestation. Increased circulating concentrations of IL-1*α*, IL-1β, IL-6, and TNF-α are consistent with previous reports describing heightened inflammatory signaling in *sarcoptic* mange (10, 74, 76–79). The inflammatory biomarker profile further indicates the activation of the innate immune response during natural mange infestation. Increased circulating concentrations of IL-1α, IL-1β, IL-6, and TNF-α are consistent with previous reports describing heightened inflammatory signaling in *sarcoptic* mange (76–79). Although these mechanisms were not directly investigated in this study, they provide a biologically plausible explanation for the observed cytokine profile. Conversely, the reduction in IL-10 may indicate diminished anti-inflammatory regulation, which could favor the persistence of inflammatory responses and the progression of skin lesions. Collectively, these findings suggest that dysregulation of the balance between pro-inflammatory and anti-inflammatory mediators is associated with the pathogenesis of ovine sarcoptic mange and may contribute to the severity of lesions. However, additional mechanistic studies evaluating local skin immune responses are required to establish causal relationships between these factors.

Correlation analysis provides additional insights into the relationship between transcriptional responses and systemic immune alterations during natural mange infestation. Rather than demonstrating causal mechanisms, these associations suggest that oxidative stress- and inflammation-related pathways are closely linked to disease progression. The positive association between *ATF4* expression and serum ceruloplasmin concentration is consistent with the coordinated activation of cellular stress response pathways reported in inflammatory conditions (80, 81). As both molecules are involved in maintaining redox homeostasis, their concurrent upregulation may reflect an adaptive response to increased oxidative stress. However, the present study did not investigate the regulatory interactions between *ATF4* and ceruloplasmin; therefore, this interpretation should be considered hypothesis-generating. The inverse relationship between *IL33* expression and circulating pro-inflammatory cytokines suggests that reduced *IL33* expression is associated with a more pronounced systemic inflammatory profile in sheep affected by mange. Previous studies have reported altered *IL-33* signaling in chronic inflammatory and parasitic diseases, although its role appears to depend on the host species, tissue type, and stage of infection (82, 83). Consequently, our findings should be interpreted as supporting a potential association between *IL33* dysregulation and inflammatory severity, rather than demonstrating a direct immunoregulatory function in ovine *sarcoptic* mange. Clarifying this relationship will require functional studies examining *IL-33* signaling within affected skin lesions.

Similarly, the negative correlations between *SLC7A11* expression and circulating inflammatory cytokines are consistent with previous evidence linking *SLC7A11*-mediated cystine transport and glutathione synthesis to the regulation of cellular redox balance and inflammatory signaling (84). Animals with relatively higher *SLC7A11* expression tended to exhibit lower systemic inflammatory responses, suggesting that the preservation of antioxidant capacity may be associated with a reduced inflammatory burden. However, these findings are based on observational correlations and do not establish that reduced *SLC7A11* expression directly promotes inflammation. Further mechanistic studies are needed to determine whether altered *SLC7A11* activity contributes to the pathogenesis of *sarcoptic* mange or reflects the severity of the inflammatory response. Although several correlations exhibited very high absolute correlation coefficients, these estimates should be interpreted with caution because they were derived from a relatively small sample set used for the gene expression analysis. Small sample sizes can yield unstable or inflated Pearson correlation coefficients; therefore, these associations should be regarded as hypothesis-generating rather than definitive. However, validation in larger independent cohorts is needed to confirm these relationships.

This study had several limitations. First, its cross-sectional design identifies associations but does not establish causal relationships between mange infestation and the observed clinical, biochemical, immunological, and molecular changes. Second, the study was conducted on naturally infected Barki sheep from a single geographic region, which may limit the generalizability of the findings to other breeds and production systems. Third, the relatively small subset of animals used for gene expression and correlation analyses may have reduced statistical precision and contributed to the high correlation coefficients observed; therefore, these findings should be considered exploratory and validated in larger animal populations. Fourth, relative gene expression was quantified using the 2^−ΔΔCt method, whereas statistical analyses were performed on ΔCt values, which are considered more appropriate for the parametric analysis of qPCR data. Consequently, although fold changes are presented to illustrate the magnitude of gene expression differences, confidence intervals for fold-change estimates were not calculated because the statistical inference was based on ΔCt values. Finally, molecular analyses were limited to selected candidate genes and were not supported by functional or protein-level validation. Future longitudinal and multicenter studies incorporating functional analyses are warranted to confirm the biological significance and clinical applicability of these findings.

## 5 Conclusion

This study investigated the genetic, transcriptional, and immune–oxidative responses of Barki ewes to sheep mange. Infected animals exhibited clinical signs alongside significant changes in APPs, oxidative stress, and inflammatory profiles, indicating the activation of innate and humoral immunity. Mange infestation was associated with altered expression of immune and antioxidant genes: upregulation of *CARD9, CLEC7A, IL33, SESN2, ATF4*, and *HSPA1A* reflected immune and stress responses, whereas reduced *TNFAIP3* and *SLC7A11* suggested impaired inflammatory control and antioxidant capacity. The detected SNPs further support a genetic contribution to susceptibility. These results highlight the role of host molecular responses in mange and suggest that immune-and oxidative stress–related genes are potential markers for resistance, warranting further validation in larger populations for genetic improvement programs.

## Data Availability

The nucleotide sequence data generated in this study have been submitted to GenBank and assigned the following accession numbers: PZ710110, PZ710111, PZ710112, PZ710113, PZ710114, PZ710115, PZ710116, PZ710117, PZ713314, and PZ713315. The original contributions presented in this study are included in the article. Further inquiries can be directed to the corresponding authors.
