# Supplementary material for: Nucleotide sequence variations, gene expression profiles, and host biomarkers associated with ovine mange caused by Sarcoptes and Psoroptes mites: implications for diagnosis and disease control
Source: Front Vet Sci. 2026 Sep 1;13:1902195. doi: 10.3389/fvets.2026.1902195 (PMC13574585; doi:10.3389/fvets.2026.1902195)
Supplement: Supplementary file 1 [file Data_Sheet_1.PDF]

```
XM_027966185.3      ATGTCCGACTACGAGAACGAGGACGCATGCTGGAGCACCCTGGAGGGCTTCCGAGTGAAG   60
H                   ATGTCCGACTACGAGAACGAGGACGCATGCTGGAGCGCCCTGGAGGGCTTCCGAGTGAAG   60
M                   ATGTCCGACTACGAGAACGAGGACGCATGCTGGAGCACCCTGGAGGGCTTCCGAGTGAAG   60
                    ************************************ ***********************

XM_027966185.3      CTCATCTCCGTCATCGACCCCGCCCGCATGACCCCCTACCTGCGCCAGTGCAAGGTGCTG   120
H                   CTCATCTCCGTCATCGACCCCGCCCGCATGACCCCCTACCTGCGCCAGTGCAAGGTGCTG   120
M                   CTCATCTCCGTCATCGACCCCGCCCGCATGACCCCCTACCTGCGCCAGTGCAAGGTGCTG   120
                    ************************************************************

XM_027966185.3      AACCCCGACGACGAGGAGCAGGTGCTCAGCGACCCCAGCCTGGTCACCCGCAAACGGAAA   180
H                   AACCCCGACGACGAGGAGCAGGTGCTCAGCGACCCCAGCCTGGTCACCCGCAAACGGAAA   180
M                   AACCCCGACGACGAGGAGCAGGTGCTCAGCGACCCCAGCCTGGTCACCCGCAAACGGAAA   180
                    ************************************************************

XM_027966185.3      GTGGGAGTGCTCCTGGACATCCTGCAGCGGACCGGCCACAAGGGCTACGTGGCCTTCCTC   240
H                   GTGGGAGTGCTCCTGGACATCCTGCAGCGGACCGGCCACAAGGGCTACGTGGCCTTCCTC   240
M                   GTGGGAGTGCTCCTGGACATCCTGCAGCGGACCGGCCACAAGGGCTACGTGGCCTTCCTC   240
                    ************************************************************

XM_027966185.3      GAGAGCCTGGAGCTGTACTACCCGCAGCTCTACAGGAAGGTCACGGGCAAGGAGCCCACC   300
H                   GAGAGCCTGGAGCTGTACTACCCGCAGCTCTACAGGAAGGTCACGGGCAAGGAGCCCACC   300
M                   GAGAGCCTGGAGCTGTACTACCCGCAGCTCTACAGGAAGGTCACGGGCAAGGAGCCCACC   300
                    ************************************************************

XM_027966185.3      CGTGTCTTCTCCATGATCATCGACGCTTCCGGTGAGTCAGGCCTGACGCAGCTGCTGATG   360
H                   CGCGTCTTCTCCATGATCATCGACGCTTCCGGTGAGTCAGGCCTGACGCAGCTGCTGATG   360
M                   CGTGTCTTCTCCATGATCATCGACGCTTCCGGTGAGTCAGGCCTGACGCAGCTGCTGATG   360
                    ** *********************************************************

XM_027966185.3      AGCGAGGT         368
H                   AGCGAGGT         368
M                   AGCGAGGT         368
                    ********
```

Figure S1. Comparative alignment of coding DNA sequence (CDS) fragments of the CARD9 gene (368 bp) between healthy and mange-infected Barki sheep relative to the corresponding reference sequence retrieved from GenBank (XM_027966185.3). H = healthy; M = mange-infected. Asterisks (*) indicate identical nucleotides.

```
XM_060413428.1      GATGGATATACTCAATTAGACTTCAGCTCTCGCAGCATCACCAGGAGATCTGTAGTCTCA    60
H                   GATGGATATACTCAATTAGACTTCAGCTCTCGCAGCATCACCAGGAGATCTGTAGTCTCA    60
M                   GATGGATATACTCAATTAGACTTCAGCTCTCGCAGCATCACCAGGAGATCTGTAGTCTCA    60
                    ************************************************************

XM_060413428.1      GAGAAAGGGCTTTGTGCTGCATCCTCTCGTTGGCGTCTGATTGCTGTGACTCTGGGCATT    120
H                   GAGAAAGGGCTTTGTGCTGCATCCTCTCGTTGGCGTCTGATTGCTGTGACTCTGGGCATT    120
M                   GAGAAAGGGCTTTGTGCTGCATCCTCTCGTTGGCGTCTGATTGCTGTGACTCTGGGCATT    120
                    ************************************************************

XM_060413428.1      GTATGCTCAGTGCTGCTGGTGATAACTGTGGTCCTGAGTACCTTGGGTATTTGGAGATCC    180
H                   GTATGCTCAGTGCTGCTGGTGATAACTGTGGTCCTGAGTACCTTGGGTATTTGGAGATCC    180
M                   GTATGCTCAGTGCTGCTGGTGATAACTGTGGTCCTGAGTACCTTGGGTATTTGGAGATCC    180
                    ************************************************************

XM_060413428.1      AGTTCAGTTGAACTGTTGAAGAGTGACAGCTTTCCATCAAGAAATAAAGACAACCAGAGT    240
H                   AGTTCAGTTGAACTGTTGAAGAGTGACAGCTTTCCATCAAGAAATAAAGACAACCAGAGT    240
M                   AGTTCAGTTGAACTGTTGAAGAGTGACAGCTTTCCATCAAGAAATAAAGACAACCAGAGT    240
                    ************************************************************

XM_060413428.1      CAACCCACACAATCATCTTTAGAAGATAGTGTGATACCTACCAAGGCTCTCACGACCACA    300
H                   CAACCCACACAATCATCTTTAGAAGATAGTGTGATACCTACCAAGGCTCTCACGACCACA    300
M                   CAACCCACACAATCATCTTTAGAAGATAGTGTGATACCTACCAAGGCTCTCACGACCACA    300
                    ************************************************************

XM_060413428.1      GGAGTTTTCTCTAGCGCTTGTCCCCCTAACTGGATCACACGTGAGGATAGCTGTTATCTA    360
H                   GGAGTTTTCTCTAGCGCTTGTCCCCCTAACTGGATCACACGTGAGGATAGCTGTTATCTA    360
M                   GGAGTTTTCTCTAGCGCTTGTCCCCCTAACTGGATCACACGTGAGGATAGCTGTTATCTA    360
                    ************************************************************

XM_060413428.1      TTTAACACACTATTAGATTCCTGGGATGGAAGTAAAAGACGATGCTTTCAACTGGGCTCT    420
H                   TTTAACACACTATTAGATTCCTGGGATGGAAGTAAAAGACGATGCTTTCAACTGGGCTCT    420
M                   TTTAACACACTATTAGATTCCTGGGATGGAAGTAAAAGACGATGCTTTCAACTGGGCTCT    420
                    ************************************************************

XM_060413428.1      AATCTCCTGAAGATAGACAGCTCAAAAGAGTTGGCGTGTATGTATGGGATTCCCCTGGTG    480
H                   AATCTCCTGAAGATAGACAGCTCAAAAGAGTTGGCATGTATGTATGGGATTCCCCTGGTG    480
M                   AATCTCCTGAAGATAGACAGCTCAAAAGAGTTGGCGTGTATGTATGGGATTCCCCTGGTG    480
                    *********************************** ************************

XM_060413428.1      GCTCAGTGG        489
H                   GCTCAGTGG        489
M                   GCTCAGTGG        489
                    *********
```

Figure S2. Comparative alignment of coding DNA sequence (CDS) fragments of the CLEC7A gene (489 bp) between healthy and mange-infected Barki sheep relative to the corresponding reference sequence retrieved from GenBank (XM_060413428.1). H = healthy; M = mange-infected. Asterisks (*) indicate identical nucleotides.

```
XM_060419284.1      AGGCGGCAAAAGAACCAAACGAAAGTCCCAAAGCCCACCTCCAAGCCGGGCCCCGAGCTG   60
H                   AGGCGGCAAAAGAACCAAACGAAAGTCCCAAAGCCCACCTCCAAGCCGGGCCCCGAGCTG   60
M                   AGGCGGCAAAAGAACCAAACGAAAGTCCCAAAGCCCACCTCCAAGCCGGGCCCCGAGCTG   60
                    ************************************************************

XM_060419284.1      CTCCCCGGCCTGGTGCTTGGGACCTCCCGGGGGGAAGTCTGGAGTCCTGCGGAGCCTGCC   120
H                   CTCCCCGGCCTGGTGCTTGGGACCTCCCGGGGGGAAGTCTGGAGTCCTGCGGAGCCTGCC   120
M                   CTCCCCGGCCTGGTGCTTGGGACCTCCCGGGGGGAAGTCTGGAGTCCTGTGGAGCCTGCC   120
                    ************************************************* **********

XM_060419284.1      GGAGGCCCCCACTCAGCCCCTCCGACGGCACCCAGCCTCTTCCTCTTCAGCGAGACCACG   180
H                   GGAGGCCCCCACTCAGCCCCTCCGACGGCACCCAGCCTCTTCCTCTTCAGCGAGACCACG   180
M                   GGAGGCCCCCACTCAGCCCCTCCGACGGCACCCAGCCTCTTCCTCTTCAGCGAGACCACG   180
                    ************************************************************

XM_060419284.1      GCCATGAAGTGCAGGAGCCCCGGCTGCCCCTTTACCCTCAACGTGCAGCACAACGGCTTC   240
H                   GCCATGAAGTGCAGGAGCCCCGGCTGCCCCTTTACCCTCAACGTGCAGCACAACGGCTTC   240
M                   GCCATGAAGTGCAGGAGCCCCGGCTGCCCCTTTACCCTCAACGTGCAGCACAACGGCTTC   240
                    ************************************************************

XM_060419284.1      TGTGAGCGATGCCACAATGCCCGGCAGCTGCCAGCAGGCCACCCTGCGGACCCCACACGG   300
H                   TGTGAGCGATGCCACAATGCCCGGCAGCTGCCAGCAGGCCACCCTGCGGACCCCACACGG   300
M                   TGTGAGCGATGCCACAATGCCCGGCAGCTGCCAGCAGGCCACCCTGCGGACCCCACACGG   300
                    ************************************************************

XM_060419284.1      CTTCCTGACCCGAGTAAGTGCCGAGCCTGCCTCCAGGATGTCACCAGGACGTTTAATGGC   360
H                   CTTCCTGACCCGAGTAAGTGCCGAGCCTGCCTCCAGGATGTCACCAGGACGTTTAATGGC   360
M                   CTTCCTGACCCGAGTAAGTGCCGAGCCTGCCTCCAGGATGTCACCAGGACGTTTAATGGC   360
                    ************************************************************

XM_060419284.1      ATCTGCAGTACTTGCTTTAAAAGGACTACAGCCGAGGCTGCCCCGAACCTCAGCTCCAGT   420
H                   ATCTGCAGTACTTGCTTTAAAAGGACTACAGCCGAGGCTGCCCCGAACCTCAGCTCCAGT   420
M                   ATCTGCAGTACTTGCTTTAAAAGGACTACAGCCGAGGCTGCCCCGAACCTCAGCTCCAGT   420
                    ************************************************************

XM_060419284.1      GGCTCCCTGTCCTGCCACCAGCGCTCCAAGTCTGACCCCTCACAGCTGGCCCAGAGCCT    479
H                   GGCTCCCTGTCCTGCCACCAGCGCTCCAAGTCTGACCCCTCACAGCTGGCCCAGAGCCT    479
M                   GGCTCCCTGTCCTGCCACCAGCGCTCCAAGTCTGACCCCTCACAGCTGGCCCAGAGCCT    479
                    ***********************************************************
```

Figure S3. Comparative alignment of coding DNA sequence (CDS) fragments of the TNFAIP3 gene (479 bp) between healthy and mange-infected Barki sheep relative to the corresponding reference sequence retrieved from GenBank (XM_060419284.1). H = healthy; M = mange-infected. Asterisks (*) indicate identical nucleotides.

```
XM_060409448.1      ATGAAGCCTAAAATGAAGTATTCAACCACCAAAATTTCCCCAACAAAGATGAAAAGCACA   60
H                   ATGAAGCCTAAAATGAAGTATTCAACCACCAAAATTTCCCCAACAAAGATGAAAAGCACA   60
M                   ATGAAGCCTAAAATGAAGTATTCAACCACCAAAATTTCCCCAACAAAGATGAAAAGCACA   60
                    ************************************************************

XM_060409448.1      GCAGGCAAAGCCTTGGTAAAATCTCCCAAGTTGAGAAAATCCCAACAGAAGGCTGAAGAA   120
H                   GCAGGCAAAGCCTTGGTAAAATCTCCCAAGTTGAGAAAATCCCAACAGAAGGCTGAAGAA   120
M                   GCAGGCAAAGCCTTGGTAAAATCTCCCAAGTTGAGAAAATCCCAACAGAAGGCTGAAGAA   120
                    ************************************************************

XM_060409448.1      GTTTGTCAGATGTACTATATGCAGCTACGCTCCGGCCTTAAAATAGAAAAAAAGGTCTGT   180
H                   GTTTGTCAGATGTACTATATGCAGCTACGCTCCGGCCTTAAAATAGAAAAAAAGTTCTGT   180
M                   GTTTGTCAGATGTACTATATGCAGCTACGCTCCGGCCTTAAAATAGAAAAAAAGGTCTGT   180
                    ****************************************************** *****

XM_060409448.1      TACTTTAGGAAAGAAACCACTAAAAGGCATTCATCAACAGCTGAGAAGTACAAAGAGCAT   240
H                   TACTTTAGGAAAGAAACCACTAAAAGGCATTCATCAACAGCTGAGAAGTACAAAGAGCAT   240
M                   TACTTTAGGAAAGAAACCACTAAAAGGCATTCATCAACAGCTGAGAAGTACAAAGAGCAT   240
                    ************************************************************

XM_060409448.1      CTGGCACTATTTACCTGTAAGCAGACAAAACACTTTAGTCGGTCTGTGGACCAGGGCTTC   300
H                   CTGGCACTATTTACCTGTAAGCAGACAAAACACTTTAGTCGGTCTGTGGACCAGGGCTTC   300
M                   CTGGCACTATTTACCTGTAAGCAGACAAAACACTTTAGTCGGTCTGTGGACCAGGGCTTC   300
                    ************************************************************

XM_060409448.1      ACCTTGGGTAATACAACGGTCAAGAAATACTCTACAGAAAATCACCTATCAAGTATCAAA   360
H                   ACCTTGGGTAATACAACGGTCAAGAAATACTCTACAGAAAATCACCTATCAAGTATCAAA   360
M                   ACCTTGGGTAATACAACGGTCAAGAAATACTCTACAGAAAATCACCTATCAAGTATCAAA   360
                    ************************************************************

XM_060409448.1      GACAAGGTG         369
H                   GACAAGGTG         369
M                   GACAAGGTG         369
                    *********
```

Figure S4. Comparative alignment of coding DNA sequence (CDS) fragments of the IL33 gene (369 bp) between healthy and mange-infected Barki sheep relative to the corresponding reference sequence retrieved from GenBank (XM_060409448.1). H = healthy; M = mange-infected. Asterisks (*) indicate identical nucleotides.

```
XM_027965423.2      ATGATCGTTGCGGACTCCGAGTGCCGCGCGGAGCTGAAGGGCTACCTGCCCGGGGCTGGA    60
H                   ATGATCGTTGCGGACTCCGAGTGCCGCGCGGAGCTGAAGGGCTACCTGCCCGGGGCTGGA    60
M                   ATGATCGTTGCGGACTCCGAGTGCCGCGCGGAGCTGAAGGGCTACCTGCCCGGGGCTGGA    60
                    ************************************************************

XM_027965423.2      GAGGAGCAGAGAGAGAGCCGCGTCCGACGAGGCCCCCGAGGGCCCAGCGCCTTCATTCCA    120
H                   GAGGAGCAGAGAGAGAGCCGCGTCCGACGAGGCCCCCGAGGGCCCAGCGCCTTCATTCCA    120
M                   GAGGAGCAGAGAGAGAGCCGCGTCCGACGAGGCCCCCGGGGGCCCAGCGCCTTCATTCCA    120
                    ************************************** *********************

XM_027965423.2      GTAGAGGAGGTCCTGCAGGAGGGAGCCGAGAGCCTCGAGCAGCACCTGGGGCTGGAGGCA    180
H                   GTAGAGGAGGTCCTGCAGGAGGGAGCCGAGAGCCTCGAGCAGCACCTGGGGCTGGAGGCA    180
M                   GTAGGGGAGGTCCTGCAGGAGGGAGCCGAGAGCCTCGAGCAGCACCTGGGGCTGGAGGCA    180
                    **** *******************************************************

XM_027965423.2      CTGATGTCCTCGGGGCGGGTGGACAACCTGGCCGTGGTGATGGGCCTGCACCCTGACTAC    240
H                   CTGATGTCCTCGGGGCGGGTGGACAACCTGGCCGTGGTGATGGGCCTGCACCCTGACTAC    240
M                   CTGATGTCCTCGGGGCGGGTGGACAACCTGGCCGTGGTGATGGGCCTGCACCCTGACTAC    240
                    ************************************************************

XM_027965423.2      TTTACCAGCTTCTGGCGCCTGCACTACCTGCTGCTGCACACGGATGGGCCCCTGGCCAAT    300
H                   TTTACCAGCTTCTGGCGCCTGCACTACCTGCTGCTGCACACGGATGGGCCCCTGGCCAAT    300
M                   TTTACCAGCTTCTGGCGCCTGCACTACCTGCTGCTGCACACGGATGGGCCCCTGGCCAAT    300
                    ************************************************************

XM_027965423.2      TCCTGGCGCCATTACATCGCCATCATGGCTGCTGCCCGCCACCAGTGTTCCTACCTGGTG    360
H                   TCCTGGCGCCATTACATCGCCATCATGGCTGCCGCCCGCCACCAGTGTTCCTACCTGGTG    360
M                   TCCTGGCGCCATTACATCGCCATCATGGCTGCTGCCCGCCACCAGTGTTCCTACCTGGTG    360
                    ******************************** ***************************

XM_027965423.2      GGCTCCCACATGGCCGAGTTCCTGCAGACTGGCGGTGACCCTGAGTGGCTGCTTGGCCTC    420
H                   GGCTCCCACATGGCCGAGTTCCTGCAGACTGGCGGTGACCCTGAGTGGCTGCTTGGCCTC    420
M                   GGCTCCCACATGGCCGAGTTCCTGCAGACTGGCGGTGACCCTGAGTGGCTGCTTGGCCTC    420
                    ************************************************************

XM_027965423.2      CAC     423
H                   CAC     423
M                   CAC     423
                    ***
```

Figure S5. Comparative alignment of coding DNA sequence (CDS) fragments of the SESN2 gene (423 bp) between healthy and mange-infected Barki sheep relative to the corresponding reference sequence retrieved from GenBank (XM_027965423.2). H = healthy; M = mange-infected. Asterisks (*) indicate identical nucleotides.

```
XM_060411578.1      ATCGCTACCCTGGCGGTTAAGCGGATGTACGACAGGGCGATCAGCGCCCCCACCAGCCCC    60
H                   ATCGCTACCCTGGCGGTTAAGCGGATGTACGACAGGGCGATCAGCGCCCCCACCAGCCCC    60
M                   ATCGCTACCCTGGCGGTTAAGCGGATGTACGACAGGGCGATCAGCGCCCCCACCAGCCCC    60
                    ************************************************************

XM_060411578.1      ACCCGCCTGAGCCATTCGGGGAAAAGGAGCTGGGAAGAACCCAACTGGATGGGCTCTCCC    120
H                   ACCCGCCTGAGCCATTCGGGGAAAAGGAGCTGGGAAGAACCCAACTGGATGGGCTCTCCC    120
M                   ACCCGCCTGAGCCATTCGGGGAAAAGGAGCTGGGAAGAACCAAACTGGATGGGCTCTCCC    120
                    ***************************************** ******************

XM_060411578.1      CGACTGCTCAATAAGGACATGAAGGCAGGCCTGAGCAGGTCCCTGCAGGCCCTTCCCACA    180
H                   CGACTGCTCAATAAGGACATGAAGGCAGGCCTGAGCAGGTCCCTGCAGGCCCTTCCCACA    180
M                   CGACTGCTCAATAAGGACATGAAGGCAGGCCTGAGCAGGTCCCTGCAGGCCCTTCCCACA    180
                    ************************************************************

XM_060411578.1      GGCTCCTCTGCCTTCGACACAGATACATTCTGCCCGCCCCAGCCCAAGCCATTGGCCAGG    240
H                   GGCTCCTCTGCCTTCGACACAGATACATTCTGCCCGCCCCAGCCCAAGCCATTGGCCAGG    240
M                   GGCTCCTCTGCCTTCGACACAGATACATTCTGCCCGCCCCGGCCCAAGCCATTGGCCAGG    240
                    **************************************** *******************

XM_060411578.1      AAGGGCCAGGTAGACTTGAAGAAGTCACGACTCCGCATGTCCCTGCAGGAGAAACTTCTC    300
H                   AAGGGCCAGGTAGACTTGAAGAAGTCACGACTCCGCATGTCCCTGCAGGAGAAACTTCTC    300
M                   AAGGGCCAGGTAGACTTGAAGAAGTCACGACTCCGCATGTCCCTGCAGGAGAAACTTCTC    300
                    ************************************************************

XM_060411578.1      ACTTACTACCGGAACCGGGCGGCCATCCCTGCTGGCGAGCAGGCTCGGGCCAAGCAAGCT    360
H                   ACTTACTACCGGAACCGGGCGGCCATCCCTGCTGGCGAGCAGGCTCGGGCCAAGCAAGCT    360
M                   ACTTACTACCGGAACCGGGCGGCCATCCCTGCTGGCGAGCAGGCTCGGGCCAAGCAAGCT    360
                    ************************************************************

XM_060411578.1      GCTGTGGACATATGTGCCGAGCTCCGGAGCTTCCTGCGGGCCAAGTTGCCTGATATGCCA    420
H                   GCTGTGGACATATGTGCCGAGCTCCGGAGCTTCCTGCGGGCCAAGTTGCCTGATATGCCA    420
M                   GCTGTGGACATATGTGCCGAGCTCCGGAGCTTCCTGCGGGCCAAGTTGCCTGATATGCCA    420
                    ************************************************************

XM_060411578.1      CTTCGGGACATGTACCTGAGTGGCAGCCTCTATGATGAC      459
H                   CTTCGGGACATGTACCTGAGTGGCAGCCTCTATGATGAC      459
M                   CTTCGGGACATGTACCTGAGTGGCAGCCTCTATGATGAC      459
                    ***************************************
```

Figure S6. Comparative alignment of coding DNA sequence (CDS) fragments of the ATF4 gene (459 bp) between healthy and mange-infected Barki sheep relative to the corresponding reference sequence retrieved from GenBank (XM_060411578.1). H = healthy; M = mange-infected. Asterisks (*) indicate identical nucleotides.

```
NM_001267874.1      CCGGTGGTGCAGTCGGACATGAAGCACTGGCCTTTCCGCGTGATCAACGACGGAGACAAG   60
H                   CCGGTGGTGCAGTCGGACATGAAGCACTGGCCTTTCCGCGTGATCAACGACGGAGACAAG   60
M                   CCGGTGGTGCAGTCGGACATGAAGCACTGGCCTTTCCGCGTGATCAACGACGGAGACAAG   60
                    ************************************************************

NM_001267874.1      CCTAAAGTGCAGGTGAGCTACAAGGGGGAGACCAAGGCGTTCTACCCAGAGGAGATCTCG   120
H                   CCTAAAGTGCAGGTGAGCTACAAGGGGGAGACCAAGGCGTTCTACCCAGAGGAGATCTCG   120
M                   CCTAAAGTGCAGGTGAGCTACAAGGGGGAGACCAAGGCGTTCTACCCAGAGGAGATCTCG   120
                    ************************************************************

NM_001267874.1      TCGATGGTGCTGACCAAGATGAAAGAGATCGCCGAGGCGTACCTGGGCCACCCGGTGACC   180
H                   TCGATGGTGCTGACCAAGATGAAAGAGATCGCCGAGGCGTACCTGGGCCACCCGGTGACC   180
M                   TCGATGGTGCTGACCAAGATGAAAGAGATCGCCGAGGCGTACCTGGGCCACCCGGTGACC   180
                    ************************************************************

NM_001267874.1      AACGCGGTGATCACCGTGCCGGCCTACTTCAACGACTCGCAGCGGCAGGCCACCAAGGAC   240
H                   AACGCGGTGATCACCGTGCCGGCCTACTTCAACGACTCGCAGCGGCAGGCCACCAAGGAC   240
M                   AACGCGGTGATCACCGTGCCGGCCTACTTCAACGACTCGCAGCGGCAGGCCACCAAGGAC   240
                    ************************************************************

NM_001267874.1      GCGGGGGTGATCGCGGGGCTGAACGTGCTGAGGATCATCAACGAGCCCACGGCCGCCGCC   300
H                   GCGGGGGTGATCGCGGGGCTGAACGTGCTGAGGATCATCAACGAGCCCACGGCCGCCGCC   300
M                   GCGGGGGTGATCGCGGGGCTGAACGTGCTGAGGATCATCAACGAGCCCACGGCCGCCGCC   300
                    ************************************************************

NM_001267874.1      ATCGCCTACGGCCTGCACCGGACGGGCAAGGGGGAGCGCAACGTGCTCATCTTTGACCTG   360
H                   ATCGCCTACGGCCTGCACCGGACGGGCAAGGGGGAGCGCAACGTGCTCATCTTTGACCTG   360
M                   ATCGCCTACGGCCTGGACCGGACGGGCAAGGGGGAGCGCAACGTGCTCATCTTTGACCTG   360
                    *************** ********************************************

NM_001267874.1      GGCGGGGGCACGTTCGACGTGTCCATCCTGACGATCGACGACGGCATCTTCGAGGTG      417
H                   GGCGGGGGCACGTTCGACGTGTCCATCCTGACGATCGACGACGGCATCTTCGAGGTG      417
M                   GGCGGGGGCACGTTCGACGTGTCCATCCTGACGATCGACGACGGCATCTTCGAGGTG      417
                    *********************************************************
```

Figure S7. Comparative alignment of coding DNA sequence (CDS) fragments of the HSPA1A gene (417 bp) between healthy and mange-infected Barki sheep relative to the corresponding reference sequence retrieved from GenBank (NM_001267874.1). H = healthy; M = mange-infected. Asterisks (*) indicate identical nucleotides.

```
NM_001252182.1      ATTATGGAATGTATGCGTACGCCGGCTGGGTTTTATCTCAACTTTGTTACTGAAGAAGTA    60
H                   ATTATGGAATGTATGCGTACGCCGGCTGGGTTTTATCTCAACTTTGTTACTGAAGAAGTA    60
M                   ATTATGGAATGTATGCGTACGCCGGCTGGGTTTTATCTCAACTTTGTTACTGAAGAAGTA    60
                    ************************************************************

NM_001252182.1      GAAAATCCTGAAAAAACCATCCCCCTTGCAATATGTATATCCATGACCGTCGTCACTGTT    120
H                   GAAAATCCTGAAAAAACCATCCCCCTTGCAATATGTATATCCATGACCGTCGTCACTGTT    120
M                   GAAAATCCTGAAAAAACCATCCCCCTTGCAATATGTATATCCATGACCGTTGTCACTGTT    120
                    ************************************************** *********

NM_001252182.1      GGCTACGTGCTAACAAACGTGGCCTACTTCACAACCATTAGTGCCGAGGAACTGATGCTT    180
H                   GGCTACGTGCTAACAAACGTGGCCTACTTCACAACCATTAGTGCCGAGGAACTGATGCTT    180
M                   GGCTACGTGCTAACAAACGTGGCCTACTTCACAACCATTAGTGCCGAGGAACTGATGCTT    180
                    ************************************************************

NM_001252182.1      TCAAATGCAGTGGCCGTGACCTTTTCTGAGCGGCTCCTGGGAAATTTCTCATTAGCAGTT    240
H                   TCAAATGCAGTGGCCGTGACCTTTTCTGAGCGGCTCCTGGGAAATTTCTCATTAGCAGTT    240
M                   TCAAATGCAGTGGCCGTGACCTTTTCTGAGCGGCTCCTGGGAAATTTCTCATTAGCAGTT    240
                    ************************************************************

NM_001252182.1      CCGATCTTTGTTGCCCTCTCCTGCTTTGGCTCCATGAATGGTGGTGTGTTTGCTGTCTCC    300
H                   CCGATCTTTGTTGCCCTCTCCTGCTTTGGTTCCATGAATGGTGGTGTGTTTGCTGTCTCC    300
M                   CCGATCTTTGTTGCCCTCTCCTGCTTTGGCTCCATGAATGGTGGTGTGTTTGCTGTCTCC    300
                    ***************************** ******************************

NM_001252182.1      AGGTTATTCTACGTTGCATCCCGGGAGGGTCACCTTCCAGAAATCCTCTCCATGATTCAC    360
H                   AGGTTATTCTACGTTGCATCCCGGGAGGGTCACCTTCCAGAAATCCTCTCCATGATTCAC    360
M                   AGGTTATTCTATGTTGCATCCCGGGAGGGTCACCTTCCAGAAATCCTCTCCATGATTCAC    360
                    *********** ************************************************

NM_001252182.1      ATCCGGAAGCACACTCCTCTGCCAGCT        387
H                   ATCCGGAAGCACACTCCTCTGCCAGCT        387
M                   ATCCGGAAGCACACTCCTCTGCCAGCT        387
                    ***************************
```

Figure S8. Comparative alignment of coding DNA sequence (CDS) fragments of the SLC7A11 gene (387 bp) between healthy and mange-infected Barki sheep relative to the corresponding reference sequence retrieved from GenBank (NM_001252182.1). H = healthy; M = mange-infected. Asterisks (*) indicate identical nucleotides.
